# Inhibition of a novel specific neuroglial integrin signaling pathway increases STAT3-mediated CNTF expression

**DOI:** 10.1186/1478-811X-11-35

**Published:** 2013-05-21

**Authors:** Matthew P Keasey, Seong Su Kang, Chiharu Lovins, Theo Hagg

**Affiliations:** 1 Kentucky Spinal Cord Injury Research Center, Department of Neurological Surgery, University of Louisville, Louisville, KY 40292, USA; 2 Pharmacology and Toxicology, University of Louisville, Louisville, KY 40292, USA

**Keywords:** Astrocyte, Ciliary neurotrophic factor, Focal adhesion kinase, Integrin, Intercellular communication, Mice, Neurogenesis, Neuron, Pharmacological, Transcriptional gene regulation

## Abstract

**Background:**

Ciliary neurotrophic factor (CNTF) expression is repressed in astrocytes by neuronal contact in the CNS and is rapidly induced by injury. Here, we defined an inhibitory integrin signaling pathway.

**Results:**

The integrin substrates laminin, fibronectin and vitronectin, but not collagen, thrombospondin or fibrinogen, reduced CNTF expression in C6 astroglioma cells. Antibodies against αv and β5, but not α6 or β1, integrin induced CNTF. Together, the ligand and antibody specificity suggests that CNTF is repressed by αvβ5 integrin. Antibodies against Thy1, an abundant neuronal surface protein whose function is unclear, induced CNTF in neuron-astrocyte co-cultures indicating that it is a neuroglial CNTF repressor. Inhibition of the integrin signaling molecule Focal Adhesion Kinase (FAK) or the downstream c-Jun N-terminal kinase (JNK), but not extracellular regulated kinase (ERK) or p38 MAPK, greatly induced CNTF mRNA and protein expression within 4 hours. This selective inhibitory pathway phosphorylated STAT3 on its inhibitory ser-727 residue interfering with activity of the pro-transcription Tyr-705 residue. STAT3 can activate CNTF transcription because it bound to its promoter and FAK antagonist-induced CNTF was reduced by blocking STAT3. Microinjection of FAK inhibitor directly into the brain or spinal cord in adult mice rapidly induced CNTF mRNA and protein expression. Importantly, systemic treatment with FAK inhibitors over 3 days induced CNTF in the subventricular zone and increased neurogenesis.

**Conclusions:**

Neuron-astroglia contact mediated by integrins serves as a sensor to enable rapid neurotrophic responses and provides a new pharmacological avenue to exploit the neuroprotective properties of endogenous CNTF.

## Background

Endogenous CNTF regulates the development of oligodendrocytes [1] and some neurons [2], synaptic function [3], and adult CNS neurogenesis [4,5]. CNTF treatment is neuroprotective in many animal models [6-10], and promotes retinal ganglion cell regeneration [11,12] and remyelination [13]. Even so, clinical trials failed due to low penetration of CNTF into the CNS and systemic side effects after subcutaneous injections [14]. CNTF is almost exclusively expressed in the nervous system, suggesting that its pharmacological induction might solve these problems. In the CNS, CNTF is produced at very low levels primarily by astrocytes [15] but little is known about mechanisms that regulate its expression. We found that a cAMP-reducing dopamine D2 agonist induces CNTF in the brain but not the spinal cord [5], indicating the need to find more universal regulation mechanisms.

The expression of CNTF is rapidly and robustly induced in astrocytes upon brain injury [16] and stroke [17], where it serves a neuroprotective role [18], as it does in an experimental autoimmune encephalomyelitis (EAE) model [19] and the retina [20]. We found that glial CNTF is repressed by integrins and, conversely, that loss of neuron-astroglial interaction increases CNTF in vitro and in the mouse striatum after ischemic or excitotoxic neuronal loss [18].

Integrins are a group of 24 heterodimer receptors with alpha and beta subunits binding extracellular matrix (ECM) proteins as adhesion partners [21]. The neuronal ligands that bind astroglial integrins to regulate CNTF are unknown. Neurons do not make most of the classical ECM molecules although they express laminin isoforms [22,23]. Thy-1, whose function is unknown, is highly expressed by adult neurons [24] and is a ligand of αvβ3 [25] and αvβ5 integrins [26] which are expressed by astrocytes and astroglioma cells [27,28]. Integrins signal through focal adhesion kinase (FAK) which can signal downstream to the ERK, p38 and JNK pathways [29]. The intracellular signaling pathways that regulate CNTF are unknown. The transcription factor Sox-10 regulates CNTF expression in Schwann cells [30] but is not present in astrocytes [31]. IL6 and CNTF itself induce CNTF expression [12,18], suggesting a potential role of STAT3, which is downstream of their gp130 receptor [32,33].

We set out to identify the CNTF-repressing signaling pathway from neuronal ligand to astroglial transcription factor, and whether its pharmacological inhibition would increase functional CNTF using adult SVZ neurogenesis as an outcome measure.

## Results

### Glial CNTF is repressed through αvβ5 integrin

**Figure 1:**
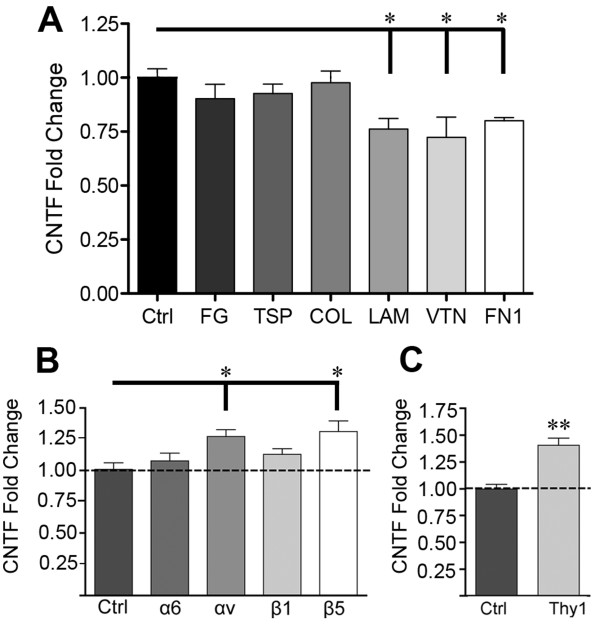
**CNTF is repressed by ECM and specific αvβ5 integrin and neuronal Thy-1 ligand. A**) C6 astroglioma cells grown for 4 h on laminin (LAM), fibronectin (FN1) and vitronectin (VTN) had lower levels of CNTF mRNA compared to poly-d-lysine control (Ctrl, set at 1.00) as quantified by qRT-PCR. Fibrinogen (FG), thrombospondin (TSP), and collagen (COL) had no significant effect. Because of the binding specificity this suggests that a limited number of integrins repress CNTF. **B**) Neutralizing antibodies against αv or β5, but not α6 or β1 induce CNTF mRNA expression relative to IgG (Ctrl) in C6 cells after 4 hours. Antibody selection was guided by elimination of unlikely candidates (Table 1). The β5 subunit is only found in αvβ5. **C**) Neutralizing antibody against Thy-1, an αvβ5 ligand, induces CNTF mRNA expression in primary astrocyte-neuron co-cultures compared to IgG (Ctrl). Data represent means (± SEM) of 3–4 (**A**) or 6 (**B**, **C**) independent experiments and are fold changes expressed relative to controls.

To identify which integrins repress CNTF, we first tested various ECM ligands with known differential integrin binding partners [21,34,35] in rat C6 astroglioma cells which express CNTF [36]. The advantage of the C6 cell is the purity, consistency and ease of the cultures compared to primary astrocytes. Moreover, the low CNTF expression by C6 cells makes them a good cell model to study changes in CNTF expression whereas the very high levels in cultured primary astrocytes combined with the half-life of 7 hours of the CNTF mRNA make it more difficult to detect modest changes under acute conditions. CNTF mRNA was decreased by ~25% when cells were cultured for 4 hours on laminin, fibronectin or vitronectin (Figure 1A). CNTF expression was not affected by fibrinogen, thrombospondin and collagen. We therefore excluded their integrin binding partners from further study (α1β1, α2β1, α3β1, α4β1, α5β1, α10β1, α11β1, and αvβ3; Table 1). We also excluded leukocyte-specific integrins from further consideration (Table 1) as well as α7, α8, β6 whose presence in astrocytes is currently unknown (Pubmed search). Finally, we did not test β8 antibodies as mature astrocytes have down-regulated αvβ8 integrin [37] and we could not obtain a suitable function-blocking antibody against rat. Having narrowed down potential integrins that might affect CNTF expression, function blocking antibodies were used against α6, αv, β1 and β5 integrin subunits. Freshly plated C6 cells incubated for 4 hours with αv and β5 integrin antibodies had 28% and 38% more CNTF mRNA, respectively, compared to no antibody or purified isotype specific IgG (Figure 1B). In contrast, α6 and β1 integrin antibodies did not significantly alter CNTF expression. Interestingly, the only integrin with a β5 subunit is αvβ5, suggesting that it may be specifically involved in inhibiting CNTF expression.

**Table 1:** Process to define which integrins repress CNTF

**Integrin**	**FG**	**TSP**	**COL**	**LAM**	**VTN**	**FN1**	**Leukocyte**	**Antibody**
α1β1			*i, ii, iii*	**i, ii, iii**				*β1*
α2β1		*i, iii*	*i, ii, iii*	**i, ii, iii**		**ii**		*β1*
α3β1		*i, ii, iii*		**i, iii**		**ii**		*β1*
α4β1		*i, iii*				**i, ii, iii**	*X*	*β1*
α4β7						**i, ii, iii**	*X*	
α5β1	*ii*	*iii*				**i, ii, iii**		*β1*
α6β1		*iii*		**i, ii, iii**				*α6, β1*
α6β4				**i, ii, iii**				*β6*
α7β1				**i, ii, iii**				*β1*
α8β1						**i, ii, iii**		*β1*
α9β1							*X*	*β1*
α10β1			*i, iii*					*β1*
α11β1			*i, ii, iii*					*β1*
αvβ1					**ii**	**i, ii, iii**		*β1*
αvβ3	*i, ii, iii*	*i, ii, iii*		ii	**i, ii, iii**	**i, ii, iii**		
αvβ5					**i, ii, iii**	**ii**		**αv, β5**
αvβ6						**i, ii, iii**		**αv**
αvβ8						**ii**		**αv**
αdβ2					**iii**	**iii**	*X*	
αLβ2							*X*	
αmβ2	*i, ii, iii*						*X*	
αxβ2	*i, ii, iii*		*iii*				*X*	
αEβ7							*X*	
αIIbβ3	*i, ii, iii*	*i, ii, iii*			**i, ii, iii**	**i, ii, iii**		

We applied the following process to help us to decide which subunit blocking antibodies to test and which integrins most likely repress CNTF. When “*italics*” appears in a row, that specific integrin is considered unlikely to be involved in repressing CNTF. We first used the data from the ECM substrate experiment (Figure 1A) and the known integrin binding partners to eliminate potential integrins involved in repressing CNTF. Thus, fibrinogen (FG), thrombospondin (TSP), and collagen (COL) had no significant effect on CNTF mRNA levels in C6 cells, as indicated by “*italics*” in the row of their known integrin partners. The numbers in the cells indicate the following articles that list the specific ligand – integrin relationship: i) Humphries et al., [34] ii) Plow et al. [38], and iii) Takada et al., [39]. Laminin (LAM), vitronectin (VTN), and fibronectin (FN) significantly reduced CNTF expression, as indicated by “**bold**” numbers in the respective columns. Next, to further help with antibody selection, we eliminated all leukocyte-specific integrins [21]) as they are not present on glial cells (second to last column). Lastly, the results from the antibody experiment (Figure 1B) were used to eliminate a number of β1- and α6-containing integrins (“*italics*” letters in the last column), leaving three candidates which potentially repress CNTF (“**bold**” letters in the last column).

### Astroglial CNTF is repressed by neuronal Thy-1

The surface protein Thy-1 is enriched in neurons throughout the CNS [40,41] and binds αvβ5 integrin [25], but its role in the brain is unknown. Primary cortical neurons were incubated with Thy-1 blocking or IgG control antibodies prior to seeding onto primary astrocyte monolayers. Thy-1 antibody increased CNTF expression by ~40% (Figure 1C). This suggests that neuronal Thy-1 is an inhibitor of astroglial CNTF expression. We did not test antibodies against laminin because the integrin binding motif is unknown. Vitronectin [42] and fibronectin [43] are not present in neurons.

### Glial Focal Adhesion Kinase represses CNTF mRNA and protein

**Figure 2:**
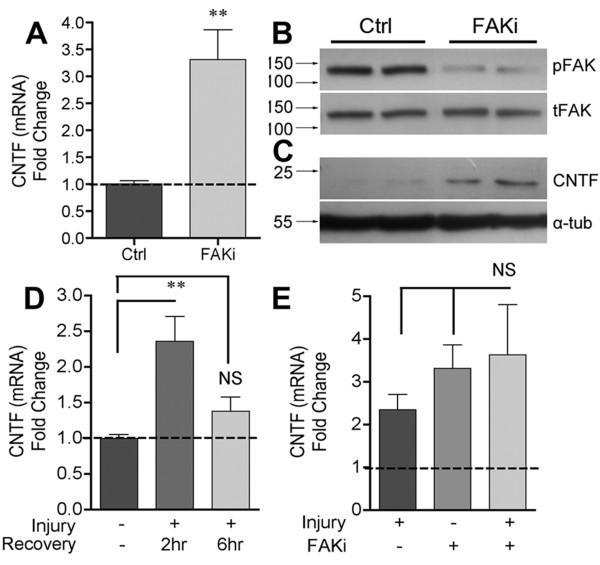
**FAK inhibition induces CNTF expression *in vitro. *A**) C6 cells incubated for 4 hours with FAK inhibitor PF573228 have increased levels of CNTF mRNA compared to vehicle-only cultures (Ctrl). **B**) The inhibitor reduced FAK activity as shown in Western blots by the reduced levels of phosphorylated FAK (pFAK tyr-397). **C**) CNTF protein was also increased in the same protein extracts, showing the rapid and robust nature of the disinhibition. Blots are representative of 4 independent observations. **D**) C6 cell injury by mechanical dissociation robustly induced CNTF mRNA within 2 hours which was lost by 6 hours. **E**) FAK inhibition did not augment the dissociation-induced CNTF mRNA up-regulation. Data are fold change compared to controls and are shown as average +/− SEM. In A, n = 6, D and E, n = 4.

FAK is the best-known kinase associated with integrin signaling [29,44]. C6 cells incubated with the FAK antagonist (FAKi) PF573228 for 4 hours showed a more than 3 fold induction of CNTF mRNA expression (Figure 2A). FAK activity was clearly decreased by the inhibitor as assessed by western blotting for phosphorylated FAK (Tyrosine-397, here on pFAK; Figure 2B). In the same protein extracts, CNTF was robustly increased by the inhibitor (Figure 2C). Wounding the C6 cells by mechanical dissociation induced CNTF expression within 2 hours (Figure 2D). CNTF mRNA levels returned to baseline after 6 hours despite similar cell survival between 2 and 6 hours (as defined by MTT assay and trypan blue counts; data not shown). This suggests that both induction and repression of CNTF occur rapidly. FAK inhibition of injured cells did not cause further increases in CNTF mRNA (Figure 2E), suggesting that modulation of FAK plays a central role in the injury-induced disinhibition of CNTF. These experiments identified FAK as a molecular target to pharmacologically increase CNTF protein expression.

### FAK-JNK activation mediates repression of CNTF

**Figure 3:**
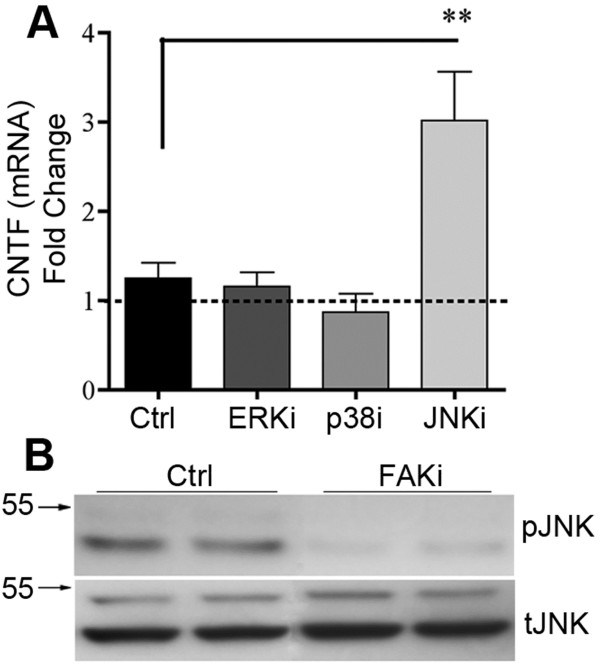
**A specific intracellular FAK-JNK signaling pathway represses CNTF. A**) Incubation of C6 cells with inhibitors of JNK, but not p38 or ERK, for 4 hours, increases CNTF mRNA compared to vehicle controls (Ctrl). Data are means +/− SEM and are from 4 independent experiments. **B**) Incubation with FAK inhibitor reduced JNK activation as shown by reduced JNK phosphorylation in western blots, which are representative of 4 independent observations. Antibodies against total JNK were used as internal controls.

Downstream targets of FAK include ERK [45], JNK [46,47] and p38 MAPK [48]. Pharmacological inhibition of JNK induced CNTF mRNA expression in C6 astroglioma cells more than 3 fold, whereas antagonists of ERK or p38 did not significantly alter CNTF expression (Figure 3A). Moreover, FAK inhibitor treatment inactivated JNK as shown by a reduction in phosphorylated JNK (pJNK) protein (Figure 3B). These data indicate that integrin-mediated CNTF repression occurs through a specific FAK-JNK signaling pathway.

### FAK represses CNTF by inhibiting STAT3 through the ser-727 residue

**Figure 4:**
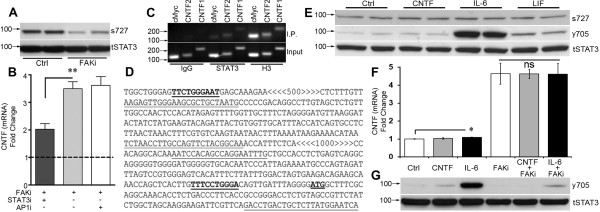
**STAT3 inhibition mediates CNTF repression by FAK. A**) FAK inhibition reduces STAT3 phosphorylation on its inhibitory residue (s727) as shown in western blots of C6 cells incubated for 4 hours. Total STAT3 antibodies were used as internal controls. **B**) Pre-incubation with a STAT3 antagonist reduced the CNTF-inducing effects of FAK inhibitor in C6 cells. Thus, FAK represses CNTF by inhibiting STAT3. An AP1 transcription factor antagonist was without effect. Data are fold compared to control (not shown) and means +/−SEM. **C**) STAT3 binds the CNTF promoter region of C6 cells as shown by ChIP analysis. STAT3 antibody immunoprecipitates (IP) CNTF promoter DNA as shown by the PCR amplification products of two promoter-specific primer sets. Normal rabbit IgG was used as control for non-specific binding. Histone H3 was a positive control. **D**) Sequencing of the amplification products of the STAT3 immunoprecipitate gave the predicted DNA regions. Key: **Consensus STAT3 binding sites**; : CNTF initiation site; CNTF1 primer set (FWD, REV); ; < > denote DNA regions excluded from this panel for presentation purposes. **E**) IL-6 treatment of C6 cells for 15 minutes robustly increased phosphorylation of STAT3 at the Tyr-705 residue (y705) with modest increases after CNTF and LIF as shown by western blot. Ser-727 phosphorylation (s727) or total STAT3 (tSTAT3) was not affected. Similar results were seen at 4 hours. The blot is representative of 4 independent experiments. **F**) IL-6 induced only an ~10% increase in CNTF mRNA expression in C6 cells after 4 hours and did not augment FAKi-induced CNTF expression (n = 3-4 each, p < 0.05). **G**) FAK inhibition reduced phosphorylation of STAT3 (y705) in C6 cells most notably under IL-6 treated conditions. Antibodies against total STAT3 were used as internal controls for western blots. Results were repeatable in independent experiments.

Activation of STAT3 transcriptional activity depends upon phosphorylation at a tyrosine residue (Tyr-705) [49,50]. STAT3 is inhibited by phosphorylation of a serine residue (Ser-727) by JNK [51]. C6 cells treated with FAK inhibitor had decreased STAT3 (Ser-727) phosphorylation (Figure 4A) in the same extracts as the reduction of JNK phosphorylation was shown (Figure 3B). Stattic is a selective inhibitor that blocks STAT3 (Tyr-705) phosphorylation, as well as STAT3 dimerization and translocation to the nucleus [52]. Incubation of stattic 1 hour prior to treatment with FAK inhibitor reduced CNTF mRNA expression 2 fold compared to FAK inhibitor alone suggesting that FAKi interferes with STAT3-stimulated CNTF expression (Figure 4B). Conversely, co-incubation with an inhibitor of the transcription factor AP-1 failed to affect FAK inhibitor induced CNTF. Our bioinformatics analyses showed that the CNTF promoter ([53]; Rat genome V3.4 Assembly; C6 cells are rat-derived) has a conserved STAT3 binding domain TTTCCTGGGA (Transcription Factor Encyclopedia, [http://www.cisreg.ca]; Motifmap, [http://motifmap.ics.uci.edu]) starting 25 nucleotides upstream of the CNTF initiation point. We also found a consensus sequence at −1954 nucleotides (TTCTGGGAA); [54]. Chromatin immunoprecipitation (ChIP) analyses in C6 cells confirmed that STAT3 binds to genomic DNA containing the CNTF promoter (Figure 4C). DNA sequencing of PCR-amplified product after the pull-down with the STAT3 antibody showed the expected CNTF gene sequence (Figure 4D).

### FAK modulates the CNTF-stimulating gp130-STAT3Tyr-705 pathway

To determine the functional relevance of a second important STAT3 phosphorylation site (Tyr-705), which is downstream of gp130-containing receptors and can stimulate cytokine expression [55-57] reviewed in [58], we incubated C6 cells with CNTF, IL-6 or LIF. Robust phosphorylation of STAT3 (Tyr-705) was observed as early as 15 minutes (Figure 4E middle row) and at 4 hours by IL-6 (~19 fold by densitometry; p < 0.001) with lesser induction by CNTF (1.6 fold, p < 0.05) and LIF (~3 fold; p < 0.01) relative to vehicle-treated control cells (data not shown). In contrast, phosphorylation of STAT3 (Ser-727) was not affected (Figure 4E, top row). These neural cytokines also did not affect total STAT3 levels (Figure 4E, lower row). Intriguingly, only IL-6 induced CNTF mRNA expression after 4 hours and only by ~10% (Figure 4F; p < 0.05). This raised the possibility that the inhibitory FAK pathway largely overrides the CNTF-stimulatory pathway and, therefore, C6 cells were treated with a combination of FAKi with CNTF or IL-6. However, IL-6 and CNTF were unable to further boost FAKi-mediated CNTF induction (Figure 4F). Finally, under the same treatment conditions, FAKi reduced phosphorylation of STAT3 (Tyr-705) most notably in the presence of IL-6 (Figure 4G), suggesting that FAK can activate STAT3 (Tyr-705), in addition to activating the inhibitory STAT3 (Ser-727).

### FAKi treatment induces CNTF and neurogenesis in the adult CNS

**Figure 5:**
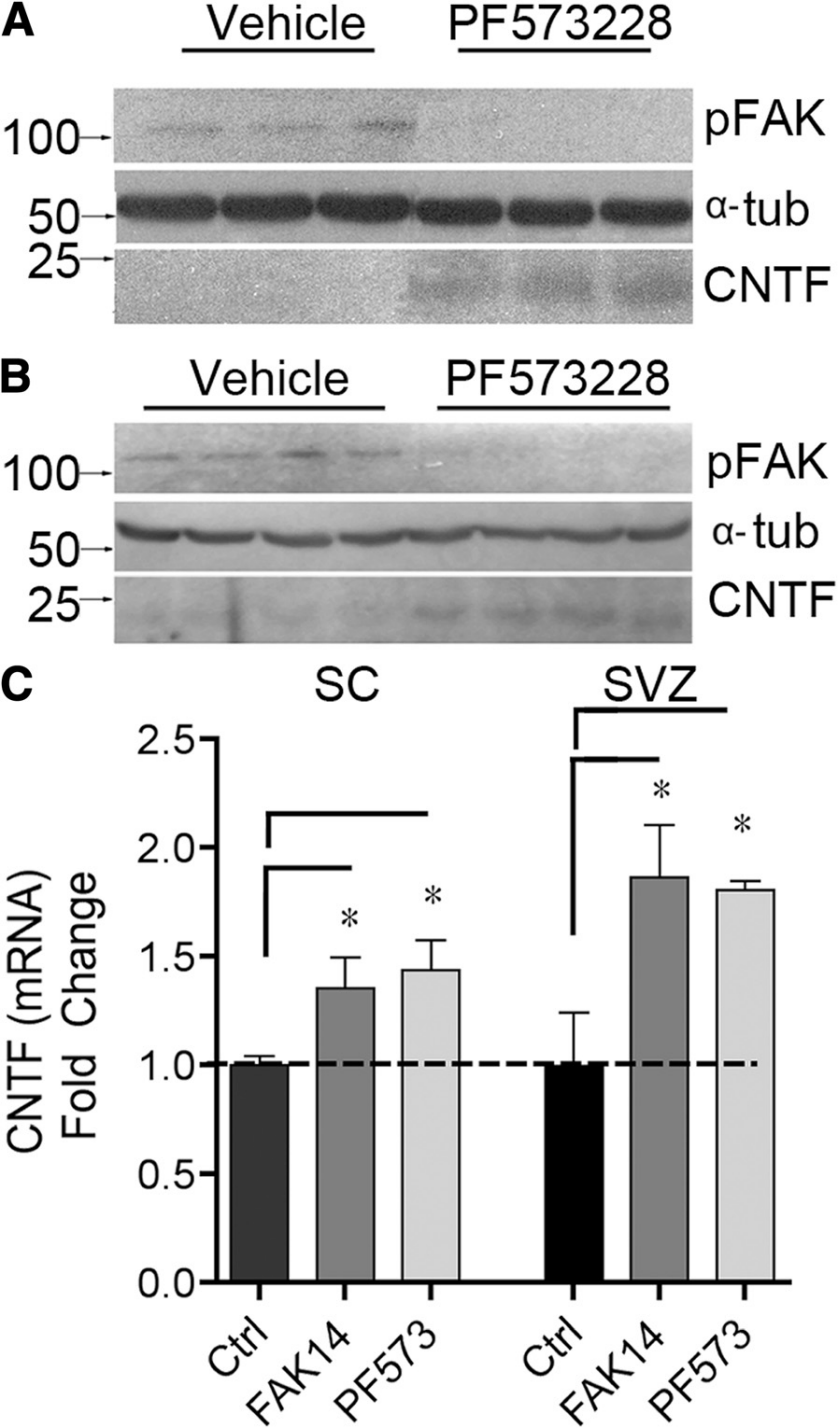
**FAK inhibition induces CNTF expression in the adult CNS *in vivo*. A**) Intracerebral injection of the FAK inhibitor PF573228 directly in the striatum of mice results in increased CNTF protein 4 hours later as shown in western blots. FAK activity was greatly decreased as shown by lack of phosphorylation. (pFAK). α-tubulin = internal control. Lanes are from individual animals. **B**) Similar results were obtained after direct injection into the spinal cord. **C**) Systemic intraperitoneal injections of the FAK inhibitors PF573228 or FAK14 induce CNTF mRNA in the spinal cord or SVZ relative to vehicle only injected mice (Ctrl). Drugs were given 3 times every 24 hours and tissues collected 2 hours after the last injection. Data are fold change compared to control (Ctrl) and are means +/− SEM from 3–4 mice per group.

The FAK inhibitor PF573228 injected directly into the adult mouse striatum or spinal cord 4 hours later caused a large decrease in pFAK (Figure 5A) and increase in CNTF protein expression (Figure 5B). Control (vehicle only) injected mice contained virtually undetectable levels of CNTF, indicating an essentially complete repression under physiological conditions and a rapid and robust increase after FAK inhibition. Separately, adult mice were injected systemically (i.p.) daily over three days with one of two FAK inhibitors. PF573228 induced CNTF mRNA ~1.8 and ~1.4 fold in the spinal cord and SVZ, respectively (Figure 5C). A second FAK inhibitor, FAK14, induced CNTF expression ~1.9 and 1.4 fold, respectively (Figure 5C).

**Figure 6:**
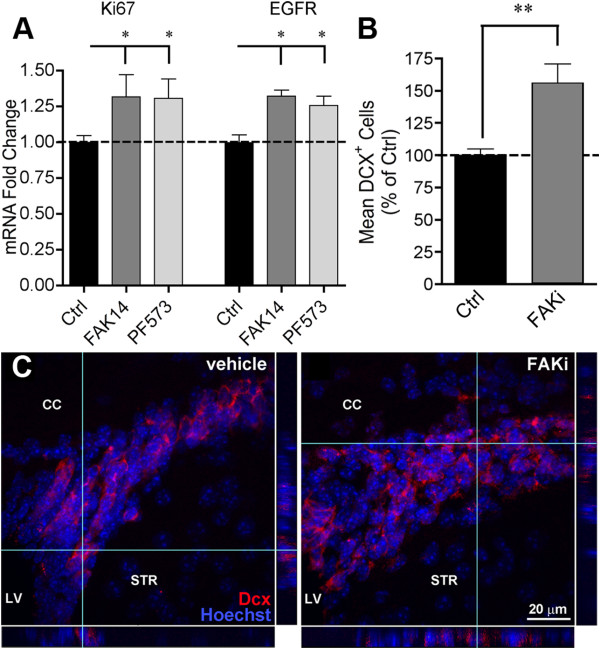
**Systemic FAK inhibitor treatment increases adult CNS neurogenesis. A**) mRNA for the proliferation marker Ki67 and the marker for proliferating neural C cell progenitors, EGFR, increased in the SVZ after systemic FAK inhibitor PF573228 treatment in mice. CNTF mRNA was increased in the same extracts (Figure 6C). Drugs were given 3 times every 24 hours and tissues collected 2 hours after the last injection. Data are mean fold change compared to vehicle treated mice +/− SEM, n = 3–4 mice. **B**) In other mice, the systemic injections of FAK inhibitor PF573228 (n = 6) increased the number of DCX + neuroblasts in the SVZ. Data are expressed as a mean percentage of vehicle (n = 4) treated mice +/− SEM. **C**) Representative confocal images of the dorsal SVZ showing increased numbers of nuclei (Hoechst) and neuroblasts (dcx) in an FAK inhibitor-injected mouse compared to a vehicle-injected mouse. Scale bar = 20 μm. CC = corpus callosum, LV = lateral ventricle, STR = striatum.

Endogenous CNTF stimulates normal neuroblast formation from the SVZ [4,5]. SVZ lysates from the mice that were injected systemically over a three day period showed that the proliferative marker Ki67 was upregulated 30% by each of the FAK inhibitors (Figure 6A). Expression of epidermal growth factor receptor (EGFR), a marker for transient amplifying progenitor SVZ cells [59], was similarly increased (Figure 6B). In another set of mice, FAK inhibitor PF573228 caused a 56% increase in the number of SVZ neuroblasts stained for their marker doublecortin (DCX; Figure 6B), confirming that neurogenesis was induced. The SVZ clearly was thicker after systemic FAK inhibitor treatment, representing more DCX cells as shown in confocal images (Figure 6C).

## Discussion

Astrocytes express a number of integrins [60] which are well-known for roles in cell morphology and adhesion [37,61-63], including αvβ5 integrin. This study identifies an αvβ5 integrin signaling pathway that regulates gene transcription, inhibiting glial CNTF expression. We cannot rule out that other integrins also repress CNTF as we did not block all integrin subunits, specifically αvβ8. However, astrocytes respond differently to vitronectin via αvβ5 and αvβ8 integrin [61], suggesting that they activate different signaling pathways. Also, adult astrocytes lack αvβ8 integrin [37]. Our data show selectivity of integrins in regulating CNTF, where blockade of αv and β5, but not α6 or β1 subunits induced CNTF expression in astroglioma cells. Cell-cell contact enables cultured astrocytes to support oligodendrocyte survival through the α6β1, but not other integrins [64]. Thus, individual integrins have specific roles for regulating gene expression.

CNTF is a member of a cytokine family, including pro-inflammatory interleukin-6 (IL-6), that also signal through the gp130 receptor [32,33]. T-cell adhesion induces IL-6 in cultured astrocytes through activation of α3β1 integrin [65]. Stretch-induced IL-6 expression in endothelial cells is mediated by α5β1 integrin [66]. Thus, two closely related cytokines are regulated by different integrins and in opposite directions, perhaps representing a mechanism by which astrocytes coordinate responses to pathological conditions. Neuronal BDNF and NGF are also upregulated by RGD-integrin signaling [67], endothelial BDNF by β1 integrins [68], and IGF-1 by α2β1 and α11β1 integrins [69]. Thus, compared to other neurotrophic factors, CNTF seems to be unique in being repressed by integrins. This explains its very low level of expression in the brain compared to other neurotrophic factors.

**Figure 7:**
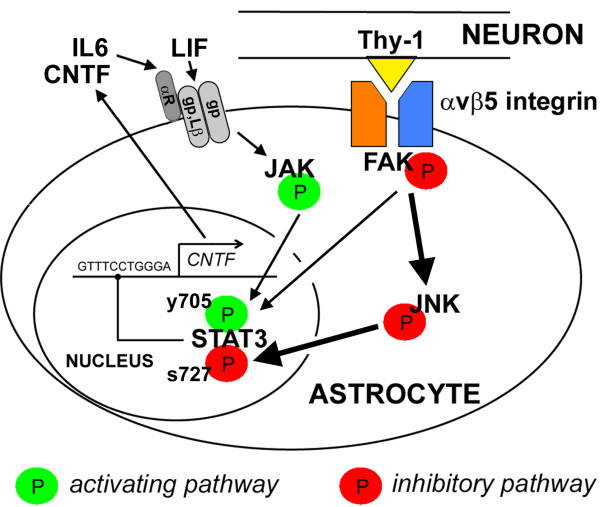
**Schematic representation of the proposed pathways that regulate astroglial CNTF.** This study identifies an inhibitory pathway (Red) where ligands such neuronal Thy-1 bind to astrocyte αvβ5 integrin resulting in FAK phosphorylation. Subsequently, JNK is activated and phosphorylates STAT3 at an inhibitory serine-727 (s727) residue potently repressing transcription of CNTF. Conversely, the CNTF-stimulatory pathway is activated by neural cytokines such as CNTF, LIF and IL-6 which bind to their respective gp130 (gp) receptor complexes (CNTF: CNTFαR/LIFβR/gp130; LIF: LIFβR/gp130; IL-6: IL-6αR/gp130/gp130; Lβ indicates LIFβR), triggering recruitment of JAK to activate STAT3 by phosphorylation at the pro-transcription residue tyrosine-705, y705; Green). CNTF is known to activate STAT3 in astrocytes in vitro [9,87-90] and in vivo [91]. FAK may simultaneously stimulate the activating pathway, potentially representing another component of the tightly regulated CNTF gene expression under physiological conditions.

Collectively, our data suggest that the CNTF-repressing integrin signaling pathway contains FAK and JNK which inhibits the transcription factor STAT3 (Figure 7). FAK promotes FGF2-induced migration of astrocytes [70] as expected from focal adhesions [71]. This study extends the role of glial FAK to gene regulation. Neurons also contain FAK [72] and in the adult, it is important for LTP [73] and plasticity [74]. FAK is largely unphosphorylated in the adult brain [75] and activated pFAK immunostaining appears highest in neurons [76]. Thus, astroglial FAK may be more responsive to inhibitors than neurons perhaps explaining why the FAK-treated mice did not have obvious behavioral changes. Clinical trials for cancer with FAK inhibitors which reach the CNS suggest that they are well-tolerated. Even so, it will be important to define the effects of chronic treatment with FAK inhibition on CNS function.

Here, JNK had a selective role in repressing CNTF whereas other major pathways downstream from FAK did not seem to be involved, i.e., ERK and p38 [48,77]. In contrast, FAK-driven JNK and ERK both regulate FGF2-induced astroglial migration [70]. The NF-kappaB pathway mediates α3β1 and α5β1 integrin stimulation of IL-6 in astrocytes [65] and endothelial cells [66]. These integrins do not regulate CNTF. Moreover, NF-kappaB is downstream of integrin linked kinase, which associates with β1 and β3 integrins [78], neither one of which regulates CNTF. Vitronectin activation of αvβ3 integrin in astrocytes signals through PKCα and RhoA, downstream of FAK [62]. However, these molecules probably do not repress CNTF as αvβ3 integrin does not either. Therefore, the JNK pathway may specifically repress CNTF, perhaps mediating the effects of vitronectin through αvβ5 but not αvβ3 integrin.

The transcription factor Sox10 is a potent positive regulator of CNTF gene transcription in Schwann cells [30]. However, in the CNS, Sox10 is specific to oligodendrocytes [79] and is not induced in reactive astrocytes [80]. It remains to be determined whether other Sox family members regulate CNTF in astrocytes. In cultured astrocytes, the CNTF promoter is also accessible to Peroxisome Proliferator-Activated Receptor gamma (PPAR-γ) in association with cAMP Response Element Binding (CREB) and Activating Transcription Factor 2 (ATF2) [81]. Induction of CNTF by these transcription factors was dependent upon nitric oxide mediated p38 MAPK activity. We propose that the gp130-JAK-STAT3 pathway is an additional pathway activating CNTF transcription in astrocytes, but that the FAK pathway chronically inhibits STAT3 at the Ser-727 residue (Figure 7), providing new insight into co-regulation by integrins and cytokine receptors. FAK inhibition robustly induced CNTF while causing a large reduction in pJNK and pSTAT3 (Ser-727), revealing a novel integrin-STAT3 link. JNK can phosphorylate STAT3 at this inhibitory site [51] and pSTAT3 (Ser-727) can have reduced transcriptional activity [82,83]. In apparent contrast, pSTAT3 (Ser-727) can cause stable STAT3-STAT3 DNA binding activity [84]. It is possible that pSTAT3 (Ser-727) has gene specific interactions similar to methyl CpG binding protein 2 which can inhibit [85] or activate transcription when associated with other transcription factors [86]. In astrocytes, CNTF induces phosphorylation of STAT3 at Tyr-705 for transcriptional activity in vitro [9,87-90] and in vivo [91]. C6 glioma cells reportedly do not express the CNTF alpha receptor [92] but can respond to CNTF [93], possibly through the IL-6 receptor to activate JAK-STAT3 (Tyr-705) signaling as shown in BaF3 cells [94]. In our hands, CNTF along with LIF only slightly activated STAT3 (Tyr-705) in C6 cells, whereas IL-6 had robust effects. This suggests that the gp130 receptor and not the LIFβR required for LIF binding, is mainly involved in regulating CNTF. The role of STAT3 is also consistent with our finding that IL-6 and CNTF increase CNTF expression in astrocytes of the adult brain [18] and that STAT3 binds the CNTF promoter. This feed-forward autoregulation by CNTF is present in the retina [12] and in astrocyte and C6 astroglioma cell cultures [36].

Despite the robust activation of STAT3 (Tyr-705) by IL-6 in C6 cells the increase in CNTF mRNA was only 10%. This suggests that the integrin-mediated inhibitor signaling “brake” is the strongest factor in determining levels of CNTF expression. In fact, IL-6 could not further increase FAKi-induced CNTF expression despite the presence of increased STAT3 (Tyr-705) compared to FAKi alone. Interestingly, FAKi reduced STAT3 (Tyr-705) phosphorylation. Identification of the intermediary signaling molecules that link FAK to STAT3 (Tyr-705) will require further study. This dual integrin-related mechanism to regulate CNTF indicates that CNTF is a highly regulated gene which is only modulated slightly under normal physiological conditions. Under pathological conditions CNTF may be greatly induced by the loss of cell-cell contact, immediately releasing the inhibitory STAT3 pathway independent of expression of cytokines, perhaps helping to make this a rapid first-responder system. The complete loss of contact, however, and therefore FAK stimulation of STAT3 (Tyr-705) might reduce the potency of other growth factors that signal through the STAT3 pathway.

Interestingly, STAT3 independently from its transcriptional function is necessary to maintain normal mitochondrial bioenergetic function, which is dependent on Ser-727 whose phosphorylated form is highly enriched in mitochondria [95,96]; reviewed in [97]. This mechanism is also present in cortical astrocytes [98]. In light of our findings, it is possible that integrin ligand binding promotes mitochondrial function through FAK-JNK-mediated STAT3 (Ser-727) phosphorylation. Whether and how the mitochondrial effects of STAT3 might affect CNTF expression remains to be determined. CNTF has also recently been found to normalize mitochondrial function in diabetic conditions [99]. This raises the possibility that under pathological conditions that reduce Ser-727 activity, CNTF expression is disinhibited in part to maintain mitochondrial function.

The function of CNTF continues to be elucidated with evidence of its role extending to stimulation of mitochondrial bioenergetic function via NF-kB signaling [99] as well as regulating neurogenesis [18] and neuroprotection [19]. With such diverse functions and as a mediator of critical protective STAT3 signaling in neurons [90], it is likely that several molecular mechanisms exist that lead to CNTF transcription.

The role of neural Thy-1 is poorly understood despite being highly enriched in the brain and exclusively present on neurons [100]. We identify Thy-1 as one of the neuronal ligands that mediates contact-dependent repression of CNTF in astrocytes. This is consistent with the finding that Thy-1 increases 100 fold during early post natal development in the CNS [24] when CNTF expression stays low, whereas it increases greatly in the peripheral nervous system during a similar time frame [15]. Thy-1 binds to astrocytic αvβ3 integrin to activate FAK resulting in morphological changes and cell-cell attachment [62,71]. Thy-1 can bind directly to αvβ5 integrin in lung fibroblasts [26], consistent with our findings that αvβ5 integrin represses CNTF and Thy-1 inhibition increases CNTF. Neuronal loss in the adult mouse brain induces CNTF within hours [18] possibly by disinhibition of Thy-1. It remains to be determined whether the other integrin substrates which inhibited CNTF expression in vitro play a similar role in the CNS. Laminin is produced by astrocytes [101] and neurons [6], vitronectin by endothelial cells [42] and fibronectin is associated with astrocytes [43].

FAK plays key roles during nervous system development [75] but its role and that of downstream JNK in adult neurogenesis had not been investigated. Importantly, inhibition of FAK with systemic drugs rapidly induced CNTF protein expression which was biologically active as suggested by the increased formation of new neuroblasts in the adult mouse SVZ. This is consistent with our findings that endogenous CNTF enhances proliferation of progenitors in the SVZ without affecting normal neuronal cell fate choice [4,5]. Our data are also consistent with the finding that SVZ neurogenesis is dependent on STAT3 [102]. Our finding that CNTF expression is higher in the SVZ than most other brain regions [5] is consistent with the absence of neuronal Thy-1 in the SVZ and RMS (Allen Brain Atlas, [http://www.brain-map.org]). This may allow CNTF-induced proliferation until the neuroblasts reach their target in the olfactory bulb which is rich in Thy-1. Integrins such as α6β1, αv and β8, and ligands such as laminin, play a key role in neuroblast migration [103-108]. Little is known about gene regulation by integrins in the SVZ. Interestingly, α6 blocking antibodies increased SVZ proliferation in vivo [106], suggesting that there is an additional growth factor which is repressed by laminin.

## Conclusion

Our data suggest that FAK inhibition rapidly induces CNTF protein expression from very low levels within four hours in vivo. This is consistent with our finding that CNTF mRNA doubles within one hour after stroke to serve a neuroprotective role [18]. Consistent with the current data, blockade of integrins with RGD peptides reduced pFAK and decreased infarct area in a rodent model of stroke [76]. We propose that this integrin-FAK pathway constitutes a sensitive neuroglial sensor for regulating neurotrophic support or neuronal function in the CNS. This study also opens up avenues for pharmacologically stimulating and utilizing the neuroprotective actions of endogenous CNTF in neurological diseases, thus circumventing the low CNS bioavailability and systemic side effects of systemic administered CNTF [14].

## Methods

All procedures involving animals were carried out in accordance with NIH guidelines and approved by the University of Louisville Institutional Animal Care and Use Committee (IACUC). Data are shown as average ± SEM.

### Cell culture

C6 astroglioma cells were obtained from ATCC and were maintained in in t75 culture flasks (USA Scientific) in DMEM supplemented with 10% Fetal Calf Serum, 1 mM L-Glutamine, 100 U Penicillin and 100 μg Streptomycin (All Gibco). Cells were passaged every three days after washing with PBS and incubation with 0.05% trypsin: Hanks Balanced Salt Solution for 2 minutes. After centrifugation, cell pellets were resuspended in fresh medium, plated at 160,000 ml^-1^ and maintained for 24 hours except where noted. C6 cells were only used between passage number 10–40.

To test effects of ECM ligands C6 cells were cultured for 4 hours on poly-d-lysine (50 μg/ml; Sigma, USA) coated multi-well culture plates (6 well, Corning) coated with vitronectin (50 μg/ml, #SRP3186), laminin (50 μg/ml, #L2020), fibronectin (50 μg/ml, #F3667), thrombospondin (0.25 μg/cm^2^, #SRP4805), fibrinogen (50 μg/ml #F3879) or collagen type I (100 μg/ml, #C5533, all Sigma) before isolation of RNA. For antibody experiments, freshly plated C6 cells were incubated with neutralizing antibodies against αv (5 μg/ml, #104108, Biolegend, UK; [109,110]), α6 (5 μg/ml, #313614, Biolegend; [111]), β1 (5 μg/ml, #102210, Biolegend; [112]) or β5 (5 μg/ml, #12-0497-41, eBiosciences, USA; [113,114]) integrins or IgG control (5 μg/ml, #402014, Biolegend) for 4 hours before isolating RNA. Pharmacological antagonists against JNK (SP600125; 25 μM; Sigma; [115]), p38 (SB203580;10 μM; Tocris; [116]), ERK (SL327, 50 μM; Tocris; [117]) or FAK (PF573228; 10 μM; Tocris; [118]) were incubated with C6 cells for 4 hours, 24 hours after initial plating. To block STAT3 activation, the selective small molecule inhibitor Stattic (10 μM; Tocris; [52]) was incubated with C6 cells 1 hour before addition of FAKi (PF573228). To block AP-1 activity C6 cells were incubated with the AP-1 antagonist SR11302 (10 μM; Tocris; [119,120]) 1 hour prior to co-incubation with FAKi (PF573228). We did not include negative controls for these inhibitors because most of the drugs used in this study are relatively well studied and elucidated and also, we do not know of the existence of inactive enantiomers for PF573228. The drugs which lacked effects on CNTF expression may serve as negative controls for the ones that did have an effect.

Primary astrocyte-neuron co-cultures were performed as described before from the cortices of neonatal C57BL/6 mice [18]. Neurons (0.5 × 10^6^/ml) were incubated with Thy-1 neutralizing antibodies (5 μg/ml; #105309, Biolegend; [121]) or isotype IgG control (5 μg/ml; #402014, Biolegend) before seeding onto the astrocytes or poly-D- lysine-coated plates. RNA was isolated after 24 hours.

### In vivo injections

Stereotaxic injection into the striatum of anesthetized mice was performed as described [18] through a glass needle with a 35 μm diameter tip attached to a pico spritzer (Parker Instrumentation) and loaded with either vehicle (75% DMSO in saline) or 20 μg PF573228 (Cat#3239, Tocris) in vehicle. One day later, the mice were transcardially perfused with ice cold PBS, the striatum dissected and flash frozen at −80°C. To inject in the spinal cord, the vertebral column was stabilized in a frame, the cord exposed with a laminectomy at thoracic level 9 and the dura incised. A volume of 1 μl containing vehicle or 20 μg PF573228 was injected into the middle of the cord. After 4 hours, mice were transcardially perfused, and a 3 mm section of cord with the injection site in the middle was dissected and flash frozen.

Systemic i.p. injections of FAK inhibitors were applied daily over three days with 30 mg/kg/day PF573228 dissolved in 100 μl of 75% DMSO or 30 mg/kg/day FAK14 (Cat#3414; Tocris), dissolved in 100 μl PBS. The brains of these mice were collected 2 hours after the last injection and processed for measuring CNTF mRNA levels. Other mice were processed for histology as described further on.

### Quantitative-RT-PCR

Total RNA was extracted from tissue and cells with the miRVana RNA isolation kit (Ambion) according to manufacturer’s protocol. RNA concentration was measured with a nano-drop Spectrophotometer. Quantitative Real Time RT-PCR (qPCR) was performed as described [18] with some minor alterations. Briefly, 0.5 μg of RNA was treated with DNAse to destroy contaminating DNA according to standard procedure. DNAse was inactivated before RNA was used to generate cDNA. Complimentary DNA was generated from 0.5 μg of RNA using MMLV reverse transcriptase (200 U), 0.5 μg random hexamers, 0.5 mM dNTP mix in a 25 μl reaction. Reactions were incubated for one hour at 37°C. The cDNA was then used with Applied Biosytems qRT-PCR primer sets specific to mouse CNTF (mM00446373_m1), GAPDH (mM4352339E), EGFR (mM00433023) and Ki67 (mM01278606) and rat primer sets were CNTF (Rn00755092) and GAPDH (Rn99999916; all Applied Biosystems). PCR reactions were performed using the TaqMan Gene Expression Master Mix (4369016, Applied Biosystems) with the following cycling parameters: 10 minutes at 95°C followed by 40 cycles of; 95°C for 15 sec; 60° for 1 minute in an ABI 7900 Thermal Cycler (Applied Biosystems). Data analysis was performed with the ΔΔCt method with GAPDH serving as an endogenous control.

### ChIP analysis

ChIP analysis was performed with the Millipore ChIP kit (#17-294, Millipore, USA) according to the manufacturer’s protocol with some minor modifications. A total of 2.56 million C6 cells were plated at 160,000 cells/ml in 75 cm^2^ flasks for 24 hours, then treated with vehicle (75% DMSO) or 10 μM FAK inhibitor PF573228 in vehicle (Cat. #3239, Tocris) for 4 hours. C6 cells were fixed with 1% formaldehyde for 10 minutes at room temperature and then washed with and resuspended in ice cold PBS supplemented with a protease inhibitor cocktail (Cat# P8340, Sigma). Cells were scraped and centrifuged at 4°C for 5 minutes at 2,000 rpm, after which the cell pellet was resuspended in 1x SDS lysis buffer and left on ice for 10 minutes. Chromatin was sheared by sonication (Misonix, XL-2000) on ice (six pulses of 10 seconds at a setting of 3) to an average size of sheared chromatin of 500 bp and up to ~1.5-2 Kbp. Sonicated samples were centrifuged for 10 minutes at 14,000 rpm at 4°C to remove any debris, and the supernatant was divided into 200 μl aliquots containing material from 1 million cells for each ChIP analysis, and then snap frozen and stored at −80°C. ChIP grade rabbit polyconal antibodies were against STAT3 (#9132, Cell Signaling, USA) or for normalization, Histone H3 (ab1791, Abcam, UK). Normal rabbit IgG (Cat#2729, Cell Signaling) was used as a control for non-specific binding. Immunoprecipitation was performed according to manufacturer’s protocol. Chromatin precipitated DNA was resuspended in a final volume of 40 ul of water (both for IP samples and input samples) and 1/10th of each was used for the PCR amplification. Primers were (Forward; Reverse): CNTF primer set 1 starting at 25 bp upstream from the CNTF initiation site (P3), 5'-AATCCACAGCCAGGAATTTG-3' and 5'-GATTCCATAAGAGCAGTCAGGTC-3', and CNTF primer set 2 starting at 1425 bp (P6), 5'-AAGAGTTGGGAAGCGCTGCTAATG-3' and 5'-TGCCGTAGAACTGGCAAGGTTAGA-3'; cMyc; a known target of STAT3 mediated transcription [122] and in C6 glioma cells [123] was 5′-GTCAACATAGCTGTACGCCCAAACGC-3′, and 5′-GTTATGTAGGAGCCCTTGCTCAGTGTG-3′. Reactions were prepared in a final volume of 20 μl with 1x PCR buffer, 200 μM dNTP, 1.5 mM MgCl^2^, 0.5 μM each forward and reverse primers, 1/10 chromatin immunoprecipitated (or Input) DNA sample and 0.5 U of Taq DNA polymerase. The PCR cycle used were 3 minutes at 94°C for the initial denaturation, 36 cycles of 45 seconds of 94°C, 30 seconds at 60°C, 60 seconds at 72°C, followed by 10 minutes at 72°C. ChIP amplification products were sequenced at the University of Louisville DNA Core Facility.

### Western blotting

Protein lysate from cell cultures was isolated using RIPA buffer (50 mM Tris–HCl, 1% [w/v] Tergitol, 0.25% [w/v] Sodium deoxycholate, 150 mM NaCl and 100 mM EDTA) supplemented with 1 mM sodium orthovanadate, 5 mM sodium fluoride and 0.1% [v/v] protease inhibitor cocktail. Cells were washed in ice cold PBS (Gibco) before cells were scraped from the surface with an inverted p1000 pipette tip in RIPA buffer. Lysate was transferred to Eppendorf tubes and placed on ice. The lysate was then triturated using a 1 ml syringe (BD) and 26½ gauge needle before samples were returned to ice and incubated for 30 minutes. Samples were centrifuged at 12,300 rpm at 4°C for 15 minutes. Lysate was then transferred to fresh Eppendorf tubes and stored at −80°C or prepared for protein quantitation with Pierce’s BCA protein assay as per manufacturer’s instructions. Proteins were separated by SDS-PAGE and blotting was then performed with specific antibodies for CNTF (1:400; MAB338, Millipore), or with FAK (1:1000; Cat#3285), pFAK-Tyr397 (1:1000; Cat#3283), JNK (1:1000; Cat#3708), pJNK-Tyr185 (1:1000; Cat#9251), pSTAT3-Ser727 (1:500; Cat#9134), pSTAT3-Tyr705 (1:1000; Cat #9145). Concentration used was 1/1000 STAT3 (1:1000; Cat#9132), αTubulin (1:2000; Cat#2125), all from Cell Signaling Technology. Briefly, after transfer, PVDF membranes were blocked in 5% non-fat milk in Tris buffered saline with 0.05% tween (TBST) for 1 hour then incubated overnight (4°C) in primary antibody (5% milk:TBST). Blots were washed with TBST before incubation (2 hours; room temperature) with appropriate Horse Radish Peroxidase conjugated secondary antibodies (anti-mouse, Cat#7076 and anti-rabbit, Cat#7074, Cell Signaling) in 5% milk; TBST. Blots were washed and ECL substrate used to visualize antibodies according to standard procedures.

### Immunocytochemistry

Mice were transcardially perfused with ice-cold PBS followed by 4% paraformaldehyde. Their brains were extracted, post-fixed overnight and cryoprotected in 30% sucrose in PB for 24 hours. Coronal 30 μm thick sections were cut on a sliding freezing microtome. Starting at a random point along the rostrocaudal axis of the brain, every sixth section through the SVZ was immunostained for doublecortin (dcx) to detect neuroblasts. Briefly, sections were incubated in 5% donkey for 1 hour followed by overnight incubation (4°C) with goat anti-DCX (1:500, SC8066, Santa Cruz). Secondary antibodies were anti-goat IgG (1:500, Alexa Fluor 488, A11055, Invitrogen) for 1 hour at room temperature. Sections were incubated with Hoechst (1 μg/ml) before cover slipping for imaging. Confocal images were taken on a Nikon D-Eclipse C1 confocal microscope. The images of 1024 × 1024 x-y pixel and 8.4 μm z-stack were taken using a 100x oil objective.

### Cell counting and statistical analysis

The number of neuroblasts was counted independently by two investigators blinded to the treatment using a 20x objective (Leica DM6000 microscope) by identifying dcx-positive cells with Hoechst-labeled nuclei in the most populated dorsal quadrant of the SVZ. Cells were counted at the same area (35 μm × 15 μm rectangular box) overlaying the entire width of the SVZ using 4 sections per brain. Statistical analyses were performed with Student’s *t* test or One Way Analysis of Variance (ANOVA) with a Dunnett’s or Bonferroni post-hoc test where noted. A value of *p* < 0.05 considered as statistically significant.

## Abbreviations

CNTF: Ciliary Neurotrophic factor; JNK: c-Jun N-terminal kinase; FAK: Focal Adhesion Kinase; ERK: Extracellular regulated kinase; MAPK: Mitogen Activated Protein Kinase; SVZ: Subventricular Zone; EAE: Experimental autoimmune encephalomyelitis; ECM: Extracellular matrix; pFAK: Phosphorylated FAK; FAKi: FAK antagonist; pJNK: Phosphorylated JNK; STAT3: Signal Transducer and Activator of Transcription 3; EGFR: Epidermal growth factor receptor; IL-6: Interleukin-6; pSTAT3: Phosphorylated STAT3; PPAR-γ: Peroxisome Proliferator-Activated Receptor gamma; CREB: Cyclic AMP Response Element Binding; ATF2: Activating Transcription Factor 2.

## Competing interests

The authors have no financial or non-financial conflicts of interest.

## Authors’ contributions

MPK, SSK, CL and TH designed and performed experiments, analyzed data and wrote the manuscript. All authors read and approved the final manuscript.
